# The Efficacy and Safety of Hepatic Arterial Infusion Chemotherapy for Mismatch Repair Proficient (pMMR)/Microsatellite Stable (MSS) Colorectal Cancer Liver Metastases (CRLM)

**DOI:** 10.1002/cam4.71663

**Published:** 2026-03-08

**Authors:** Yawei Li, Junqing Xi, Xiaoyu Huang, Yingen Luo, Xiaowu Zhang, Xiao Li

**Affiliations:** ^1^ Department of Interventional Therapy, National Cancer Center/National Clinical Research Center for Cancer/Cancer Hospital Chinese Academy of Medical Sciences and Peking Union Medical College Beijing China

**Keywords:** colorectal cancer liver metastases, efficacy, hepatic arterial infusion chemotherapy, mismatch repair proficient/microsatellite stable, safety

## Abstract

**Purpose:**

To assess the effectiveness and safety of hepatic arterial infusion chemotherapy (HAIC) in patients with mismatch repair proficient (pMMR)/microsatellite stable (MSS) colorectal cancer liver metastases (CRLM) who are resistant to standard treatments.

**Methods:**

This study retrospectively evaluated 137 consecutive patients with pMMR/MSS CRLM who underwent HAIC from July 2019 to September 2023. Progression‐free survival (PFS) was the primary outcome, with secondary outcomes being overall survival (OS), objective response rate (ORR), disease control rate (DCR), and safety. The Cox proportional hazards model was used to identify prognostic factors for survival.

**Results:**

In total, 78 patients participated, with a median age of 58 years (IQR, 50.75–64.00), and 50 were male. Among these, 28 were treated with a combination of HAIC and targeted therapy, whereas 50 were given HAIC monotherapy. For all patients, the median PFS and OS were 5.10 months (95% CI: 2.85, 7.35) and 16.80 months (95% CI: 13.07, 20.53), respectively. The ORR and DCR for intrahepatic lesions were 1.37% and 58.9%, respectively. All lesions had an ORR of 2.74% and a DCR of 30.14%. The 1‐year OS rate was 67.63 (95% CI, 57.22, 79.91). Patients undergoing HAIC, whether with or without targeted therapy, showed no significant differences in ORR and DCR. Multivariable analysis showed that the combination of HAIC and targeted therapy was not an independent risk factor for PFS and OS. No adverse events of grade 4 or higher were observed.

**Conclusion:**

HAIC shows effectiveness and tolerance in pMMR/MSS CRLM patients who are refractory to systemic therapy. However, the additive value of targeted therapy for HAIC in these patients needs to be further investigated.

In the United States, colorectal cancer (CRC) is the third most frequently diagnosed cancer and the third highest cause of cancer deaths [1]. The incidence rate of colorectal cancer ranks second among malignant tumors in China, and the mortality rate ranks fourth. Since 2000–2016, the morbidity and mortality rates of CRC have continued to increase in China [2]. Live metastasis is a common occurrence in CRC, with around half of thepatients developing such metastases during their illness [3]. Colorectal cancer liver metastases (CRLM) is the main cause of death in patients with CRC [4]. Without treatment, patients with CRLM have a median overall survival of just 6–9 months [5].

Patients with pMMR/MSS CRLM account for 95% of patients with colorectal cancer. However, these patients showed only modest response from immune checkpoint inhibitors (ICIs). The benefits of ICIs were limited to a small group (about 5%) of CRLM patients with microsatellite instability‐high (MSI‐high) or deficient mismatch repair (dMMR) tumors. Since the publication of the KEYNOTE‐177 study, immune checkpoint inhibitors have become the standard first‐line treatment for patients with MSI‐H/dMMR mCRC [6, 7]. At present, although some drugs (e.g., regorafenib, fruquintinib) have shown survival benefits in third‐line treatment of mCRC, the efficacy in pMMR/MSS CRLM patients is still limited, and there is no standard treatment after failure of second‐ or third‐line therapy. The median OS of best supportive care was only 4.6 months [8, 9, 10]. Recently, there has been an increasing interest in exploring other treatment options for this subgroup of patients. Several studies have backed the effectiveness of HAIC in CRLM patients who did not respond to standard treatment [11, 12, 13, 14]. However, there were no studies on HAIC therapy independently for patients with pMMR/MSS CRLM.

Hepatic arterial infusion chemotherapy (HAIC) can directly kill tumor cells in liver metastases through high‐concentration local chemotherapeutic drugs. Several studies have reported that HAIC is an effective and safe treatment for patients with CRLM. A few studies have reported an OS of 7.7–19.0 months with HAIC in patients with CRLM who are refractory to standard system therapy [15, 16]. Targeted drugs (such as bevacizumab, an anti‐VEGF drug, and cetuximab, an anti‐EGFR drug) can specifically block key pathways for tumor growth. Anti‐VEGF drugs can inhibit tumor neovascularization, reduce tumor blood supply, and make tumor cells more vulnerable to chemotherapy due to ischemia and hypoxia [17]. However, there are currently few studies on HAIC for patients with pMMR/MSS CRLM who are refractory to standard therapy. This study focused on assessing the effectiveness and safety of HAIC in pMMR/MSS CRLM patients who do not respond to systemic treatment.

## 1 Methods

### 1.1 Study Designs and Participants

**FIGURE 1:**
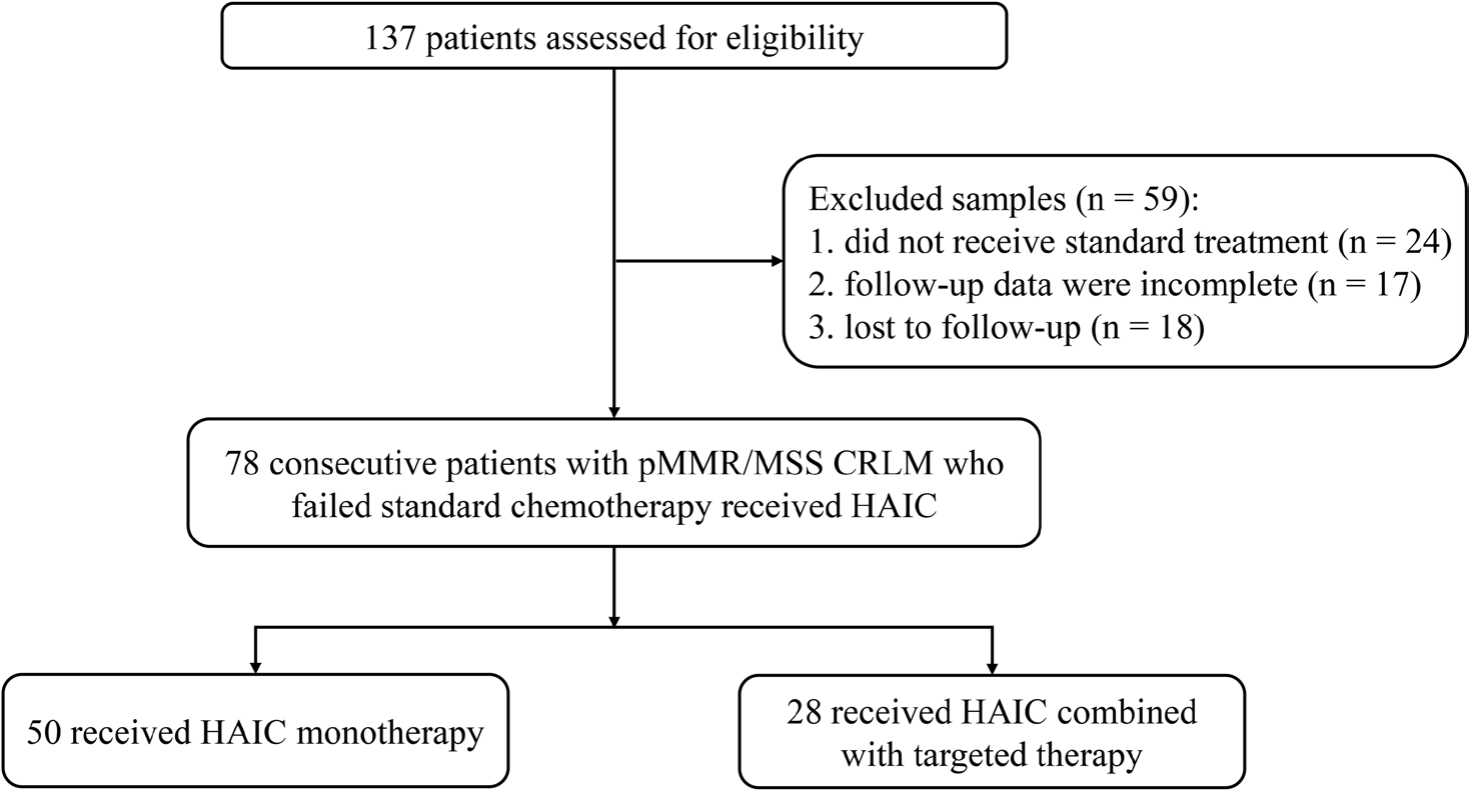
Patient enrollment flow diagram of pMMR/MSS CRLM patients treated with HAIC after standard chemotherapy failure.

A retrospective study was carried out, involving 137 consecutive patients with pMMR/MSS CRLM who underwent HAIC at the National Cancer Center, Chinese Academy of Medical Sciences and Peking Union Medical College between July 2019 and September 2023. All the patients enrolled had colorectal liver metastases and did not respond to standard first‐ and second‐line treatments. Data on baseline characteristics were extracted from the electronic medical records. The following were the inclusion criteria: pathologically confirmed pMMR/MSS CRLM and progressed on prior systemic therapies; at least one cycle of HAIC; baseline imaging within 1 month prior to HAIC. Patients were excluded from this analysis if follow‐up data were incomplete or did not receive standard first‐ or second‐line systemic therapy prior to HAIC (Figure 1). The last follow‐up time was December 31, 2023. Ethical approval was obtained from the Hospital Ethics Committee and Institutional Review Board.

### 1.2 Hepatic Arterial Infusion Chemotherapy

HAIC was implemented via the Seldinger technique. At the outset, a 5F Right Hepatic catheter was utilized to determine the quantity of liver tumors, their vascular supply, anatomical position, and related tumor arteries. This necessitated angiography of the abdominal artery, common hepatic artery, and superior mesenteric artery. Subsequently, a 2.7F microcatheter (Boston Company, USA) was directed to the left hepatic artery, right hepatic artery, or proper hepatic artery, contingent upon the tumor's distribution and blood supply.

The treatment regimens encompassed oxaliplatin, 5‐fluorouracil (5‐FU), and leucovorin (LV) (FOLFOX); irinotecan, 5‐FU, and LV (FOLFIRI); oxaliplatin, irinotecan, 5‐FU, and LV (FOLFOXIRI); as well as other options. The intra‐arterial chemotherapy protocol entailed administering 85 mg/m^2^ of oxaliplatin over 2 h, succeeded by 1000 mg/m^2^ of 5‐FU over 22 h on days 1 and 2. Moreover, irinotecan was infused at a dosage of 120 mg/m^2^ over 4 h. HAIC was administered at intervals of 4–5 weeks. The targeted drugs are administered orally according to the instructions.

### 1.3 Outcomes Assessment

The main result measured was progression‐free survival (PFS), defined as the duration from the initial HAIC to either tumor progression or death from any cause. Secondary results included OS, objective response rate (ORR), disease control rate (DCR), and safety. Tumor response was assessed by contrast‐enhanced computed tomography (CT) or magnetic resonance imaging (MRI) every 2 cycles of HAIC (approximately 8–10 weeks) according to the Response Evaluation Criteria in Solid Tumor (RECIST) version 1.1. The ORR was the rate of patients who had a complete response (CR) or partial response (PR). DCR was the rate of patients with CR, PR, and stable disease (SD). OS was the period from the first HAIC to death from any cause. AEs were assessed using the National Cancer Institute Common Toxicity Criteria, 5th edition.

### 1.4 Statistical Analysis

SPSS (version 19; SPSS, Chicago, IL, United States) and R (version 4.2.2) were used for statistical analyses. Continuous variables following a normal distribution were represented as mean ± standard deviations, while those with a skewed distribution were shown as medians (IQR). The expression of categorical variables was in the form of *n* (%). The Kaplan–Meier method was used for survival analysis, and comparisons were made using the log‐rank test. The Cox proportional hazard models were used to analyze prognostic factors. Factors with *p* < 0.1 in the univariable analyses were further included in the multivariable Cox analyses. *p* < 0.05 was considered statistically significant.

## 2 Result

### 2.1 Patient Characteristics

**FIGURE 2:**
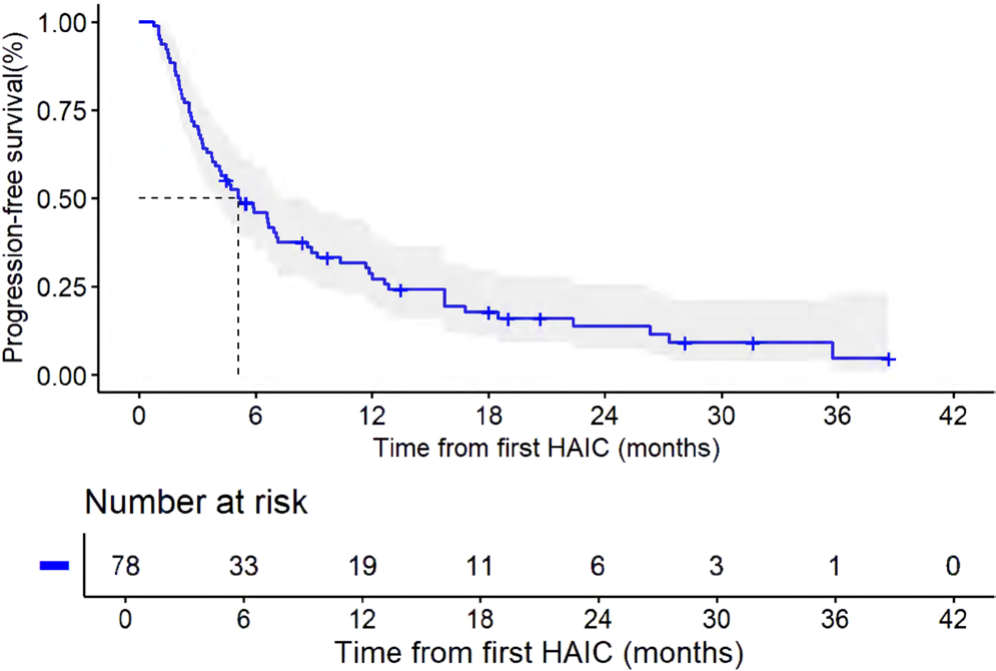
Kaplan–Meier curves for progression‐free survival (PFS) from first HAIC.

A total of 78 patients were finally included in the study (Figure 2). The median age was 58 years (IQR, 50.75–64.00 years), with 50 male (64.10%) and 28 female (35.90%). All enrolled patients experienced disease progression following the receipt of at least two lines of standard systemic treatment. Of these patients, 50 cases (64.1%) had undergone three or more lines of treatment. Among these patients, 28 received HAIC combined with targeted therapy, while 50 received HAIC monotherapy. The combination group had a significantly higher proportion of patients receiving FOLFOX regimen (*n* = 20, 71.43%), the maximum diameter exceeding 3 cm (*n* = 23, 82.14%), and more than 3 tumors (*n* = 27, 96.43%), compared to the HAIC group. The patient demographics, tumor characteristics and treatment details were summarized in Table 1.

**TABLE 1:** Baseline characteristics.

Variable	Total	HAIC	HAIC plus targeted therapy	*p*
Age (years)	58.00 (50.75, 64.00)	56.50 (50.00, 63.00)	60.50 (55.25, 64.00)	0.175
Gender				
Male	50 (64.10)	30 (60.00)	20 (71.43)	0.313
Female	28 (35.90)	20 (40.00)	8 (28.57)	
Smoking				
Yes	14 (17.95)	7 (14.00)	7 (25.00)	0.225
No	64 (82.05)	43 (86.00)	21 (75.00)	
Drinking				
Yes	16 (20.51)	10 (20.00)	6 (21.43)	0.881
No	62 (79.49)	40 (80.00)	22 (78.57)	
BMI				
Below normal weight	5 (6.41)	3 (6.00)	2 (7.14)	0.854
Normal weight	49 (62.82)	33 (66.00)	16 (57.14)	
Overweight	21 (26.92)	12 (24.00)	9 (32.14)	
Obesity	3 (3.85)	2 (4.00)	1 (3.57)	
ECOG score				
0	61 (78.21)	42 (84.00)	19 (67.86)	0.098
1	17 (21.79)	8 (16.00)	9 (32.14)	
Clinical stage				
Tx	9 (11.54)	5 (10.00)	4 (14.29)	0.798
T0	0 (0)	0 (0)	0 (0)	
T1	2 (2.56)	1 (2.00)	1 (3.57)	
T2	2 (2.56)	1 (2.00)	1 (3.57)	
T3	19 (24.36)	14 (28.00)	5 (17.86)	
T4	16 (20.51)	9 (18.00)	7 (25.00)	
Unknown	30 (38.46)	20 (40.00)	10 (35.71)	
Location of primary lesion				
Left colon	42 (53.85)	25 (50.00)	17 (60.71)	0.588
Right colon	11 (14.10)	7 (14.00)	4 (14.29)	
Rectum	25 (32.05)	18 (36.00)	7 (25.00)	
Time to metastases				
Synchronous	59 (75.64)	40 (80.00)	19 (67.86)	0.231
Metachronous	19 (24.36)	10 (20.00)	9 (32.14)	
Previous treatment lines				
2	28 (35.90)	16 (32.00)	12 (42.86)	0.588
3	27 (34.62)	19 (38.00)	8 (28.57)	
4	23 (29.48)	15 (30.00)	8 (28.57)	
Repeated times of HAIC				
1	26 (33.33)	16 (32.00)	10 (35.71)	0.521
2–3	39 (50.00)	25 (50.00)	14 (50.00)	
4–6	10 (12.82)	8 (16.00)	2 (7.14)	
> 6	3 (3.85)	1 (2.00)	2 (7.14)	
Treatment regimens				
FOLFOX	47 (60.26)	27 (54.00)	20 (71.43)	0.038
Irinotecan	10 (12.82)	10 (20.00)	0 (0.00)	
FOLFIRI	7 (8.97)	5 (10.00)	2 (7.14)	
FOLFOXIRI	1 (1.28)	0 (0.00)	1 (3.57)	
Others	13 (16.67)	8 (16.00)	5 (17.86)	
Number of intrahepatic lesions				
≤ 3	16 (20.51)	15 (30.00)	1 (3.57)	0.021
4–7	34 (43.59)	19 (38.00)	15 (53.57)	
≥ 8	28 (35.90)	16 (32.00)	12 (42.86)	
Maximum diameter of intrahepatic lesions				
≤ 3	23 (29.49)	18 (36.00)	5 (17.86)	0.029
4–5	32 (41.03)	15 (30.00)	17 (60.71)	
≥ 6	23 (29.49)	17 (34.00)	6 (21.43)	
Location of tumor metastasis				
Lung	5 (6.41)	2 (4.00)	3 (10.71)	0.541
Lymph nodes	5 (6.41)	3 (6.00)	2 (7.14)	
Lung + Lymph nodes	2 (2.56)	2 (4.00)	0 (0.00)	
Lung + Others	1 (1.28)	1 (2.00)	0 (0.00)	
Others	1 (1.28)	0 (0.00)	1 (3.57)	
No	64 (82.05)	42 (84.00)	22 (78.57)	
Immunity therapy prior to HAIC				
Yes	15 (19.23)	9 (18.00)	6 (21.43)	0.712
No	63 (80.77)	41 (82.00)	22 (78.57)	
Surgery on primary tumor				
Yes	52 (66.67)	37 (74.00)	15 (53.57)	0.066
No	26 (33.33)	13 (26.00)	13 (46.43)	
Other local treatment				
Yes	13 (16.67)	9 (18.00)	4 (14.29)	0.466
No	65 (83.33)	41 (82.00)	24 (85.71)	
Tumor markers				
CA199	41.00 (17.80, 200.70)	31.52 (15.80, 200.70)	63.56 (26.50, 214.20)	0.266
CA724	4.30 (2.28, 20.20)	4.23 (2.22, 20.33)	4.50 (2.70, 19.09)	0.487
CEA	24.67 (4.15, 109.00)	17.65 (3.22, 112.00)	35.70 (6.95, 98.88)	0.349

*Note:* Baseline characteristics of the study population (data are median [IQR] or *n* [%]).

### 2.2 Clinical Outcomes

**FIGURE 3:**
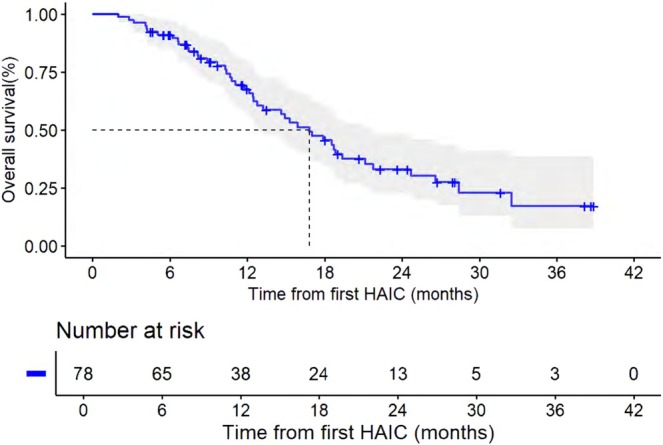
Kaplan–Meier curves for overall survival (OS) from first HAIC.

**FIGURE 4:**
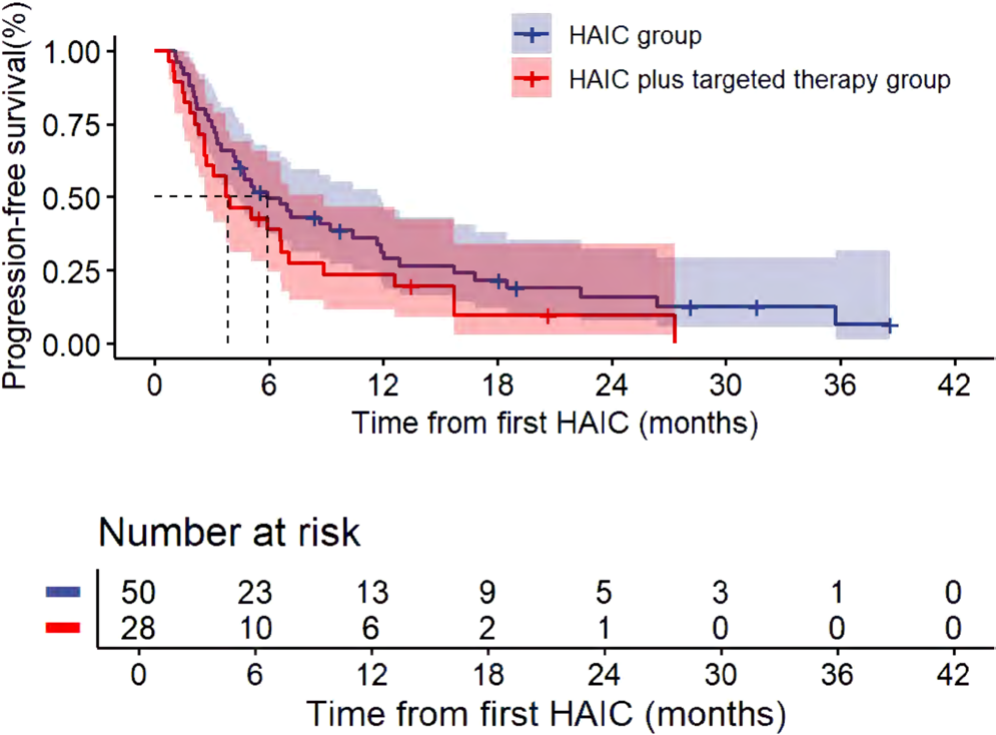
Progression‐free survival (PFS) comparison between HAIC monotherapy and HAIC plus targeted therapy groups from first HAIC.

**FIGURE 5:**
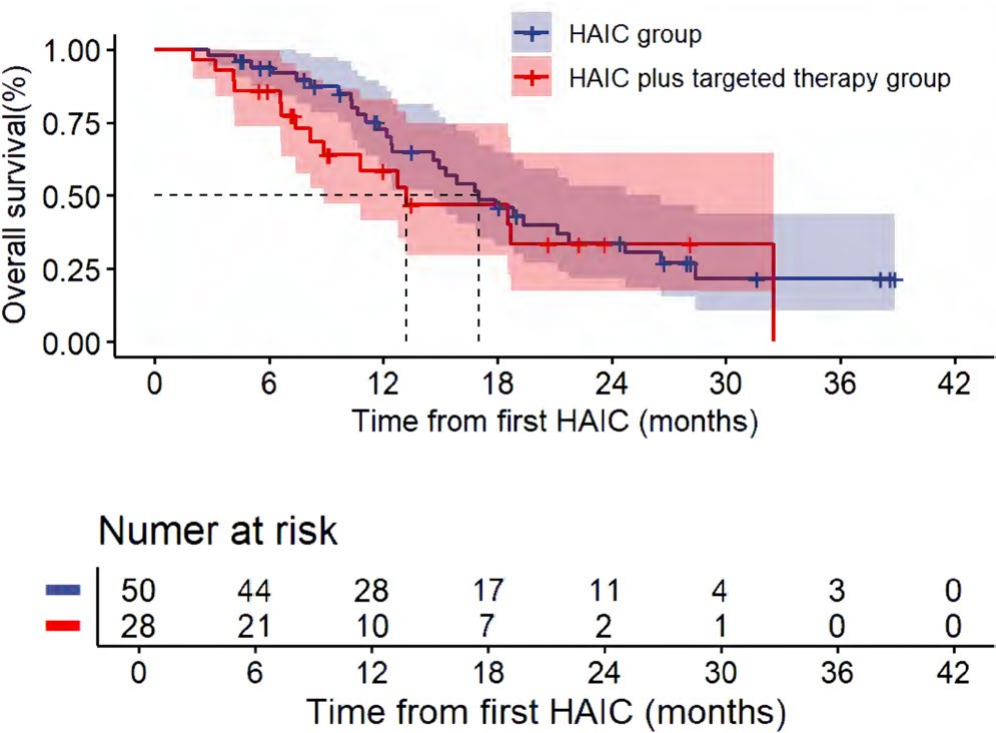
Overall survival (OS) comparison between HAIC monotherapy and HAIC plus targeted therapy groups from first HAIC.

The median follow‐up time was 24.40 (IQR: 18.27–30.53) months. For all patients, the median PFS and OS were 5.10 months (95% CI, 2.85, 7.35) and 16.80 months (95% CI, 13.07, 20.53), respectively (Figures 2 and 3). For liver tumor response rate assessment, PR was observed in 1, SD in 42, and PD in 30 patients (Table 2), achieving an ORR and DCR of 1.4% and 58.9%, respectively. For overall response rate assessment, PR was observed in 2, SD in 20, and PD in 51 patients, achieving an ORR and DCR of 2.7% and 30.1%, respectively. Patients who received HAIC with or without targeted therapy showed no statistically significant difference in ORR, DCR, PFS, and OS (Table 3, Figures 4 and 5). The rate of 1‐year OS was 67.63 (95% CI, 57.22, 79.91).

**TABLE 2:** Tumor response evaluation.

Efficacy evaluation	Intrahepatic lesions evaluation [*n* (%)]	Total lesions evaluation [*n* (%)]
CR	0 (0)	0 (0)
PR	1 (1.37)	2 (2.74)
SD	42 (57.53)	20 (27.40)
PD	30 (41.10)	51 (69.86)
ORR	1 (1.37)	2 (2.74)
DCR	43 (58.90)	22 (30.14)

*Note:* Objective response was defined as a partial or complete response. Disease control was defined as a partial, complete response and stable disease (5 patients could not be evaluated due to lack of postoperative imaging).

**TABLE 3:** Tumor response evaluation for HAIC plus targeted drug or not.

Variable	HAIC	HAIC plus	*p*
Intrahepatic lesions ORR	1 (2.22)	0 (0.00)	1.000
Intrahepatic lesions DCR	28 (62.22)	15 (53.57)	0.625
Total lesions ORR	1 (2.22)	1 (3.57)	1.000
Total lesions DCR	15 (33.33)	7 (25.00)	0.601

*Note:* Objective response was defined as a partial or complete response. Disease control was defined as a partial, complete response and stable disease.

### 2.3 Prognostic Factors for PFS and OS

In univariable analyses, factors including HAIC combined with targeted therapy, treatment regimens, number of lesions, maximum diameter of lesions, and treatment of primary lesions showed significant statistical difference (*p* < 0.1). In the multivariate analysis, these factors were considered (Table 4). Multivariable analysis identified irinotecan as an independent prognostic factor for OS. However, the combination of HAIC with targeted therapy was not observed to be an independent prognostic factor for either PFS (HR: 1.004, 95% CI: 0.556–1.813, *p* = 0.991) or OS (HR: 0.518, 95% CI: 0.238–1.127, *p* = 0.097).

**TABLE 4:** Multivariables cox regression models for PFS and OS.

Variable	Multivariable PFS			Multivariable OS		
	HR	95% CI	*p*	HR	95% CI	*p*
HAIC plus						
Without	1			1		
Plus	1.004	0.556–1.813	0.991	0.518	0.238–1.127	0.097
Treatment regimens						
FOLFOX + FOLFIRI + FOLFOXIRI	1			1		
Irinotecan	0.189	0.046–0.784	0.022	0.070	0.005–0.900	0.041
Others	0.871	0.433–1.755	0.700	0.516	0.189–1.405	0.195
Number of intrahepatic lesions						
≤ 3	1			1		
4–7	0.762	0.264–2.198	0.615	1.604	0.354–7.267	0.540
≥ 8	0.767	0.271–2.171	0.617	2.319	0.500–10.746	0.282
Maximum diameter of intrahepatic lesions						
≤ 3	1			1		
4–5	1.119	0.584–2.142	0.735	1.774	0.760–4.145	0.185
> 6	1.009	0.453–2.248	0.982	1.292	0.486–3.434	0.607
Surgery on primary tumor						
No	1			1		
Yes	0.838	0.474–1.483	0.545	0.553	0.263–1.166	0.120

*Note:* Multivariable Cox regression models for progression‐free survival (PFS) and overall survival (OS).

### 2.4 Safety

AEs related to HAIC were shown in Table 5. AEs that were grade 3 including decreased platelet count (3.85%), increased alanine aminotransferase (1.28%), and increased aspartate aminotransferase (1.28%). No grade 4 or higher AEs were observed.

**TABLE 5:** Adverse events.

Item	Grade 1	Grade 2	Grade 3	Grade 4
Pain	11 (14.10)	20 (25.64)	0 (0)	0 (0)
Vomiting	29 (37.18)	0 (0)	0 (0)	0 (0)
Fever	5 (6.41)	0 (0)	0 (0)	0 (0)
Thrombocytopenia	3 (3.85)	3 (3.85)	3 (3.85)	0 (0)
Increased aspartate aminotransferase	7 (8.97)	10 (12.82)	1 (1.28)	0 (0)
Increased alanine aminotransferase	1 (1.28)	1 (1.28)	1 (1.28)	0 (0)
Decreased albumin	56 (71.79)	10 (12.82)	0 (0)	0 (0)
Leukocytopenia	0 (0)	0 (0)	0 (0)	0 (0)

*Note:* Adverse events related to hepatic arterial infusion chemotherapy (graded by NCI‐CTC version 5.0).

## 3 Discussion

This research assessed the effectiveness and safety of HAIC for treating patients with pMMR/MSS CRLM. The median PFS and OS were 5.10 and 16.80 months, respectively. Moreover, subgroup analysis was performed on patients receiving HAIC or HAIC in combination with targeted therapy. However, there were no significant differences in ORR, PFS, and OS between these two groups. The rate of HAIC‐related grade 3 AEs was 6.4%. In our study, most of our enrolled patients had heavy liver tumor burden and developed disease progression after at least 2–3 prior standard systemic therapies, so there was a lower ORR. To sum up, this real‐world study showed good effectiveness and tolerance of HAIC in the treatment of patients with pMMR/MSS CRLM. HAIC delivered chemotherapy drugs directly to the tumor through arteries; the local drug concentration was higher, even if most patients were in the chemotherapy‐refractory stage. In a European multicenter phase II trial, the aim was to determine whether hepatic artery infusion (HAI) combined with triplet chemotherapy (consisting of irinotecan at a dose of 180 mg/m^2^, oxaliplatin at 85 mg/m^2^, and 5‐fluorouracil at 2800 mg/m^2^) and systemic cetuximab could increase the relevant rate to 30% in previously treated patients. The results showed that the ORR was 40.6% (28.6–52.3), and the median PFS and OS reached 9.3 months (7.8–10.9) and 25.5 months (18.8–32.1), respectively [18]. In another retrospective study aimed to explore the significance of hepatic arterial infusion (HAI) in patients with liver metastases (LM) from metastatic colorectal cancer (mCRC) who have become unresponsive to all standard chemotherapy regimens. For patients who had liver metastases alone, the response rate was 33%, with a median survival of 20 months. In a secondary analysis, the response rate for those with only liver metastases was 29%, and the median survival was 17.2 months [19]. It may be because these patients had experienced multiple lines of chemotherapy and targeted therapies before that and had developed drug resistance. At present, there have been some studies on combined molecular targeted therapy with immune checkpoint inhibitors in the treatment of patients with pMMR/MSS CRLM.

Based on the mechanism that targeted drugs (such as anti‐angiogenic drugs) can improve the tumor microenvironment and enhance the delivery of chemotherapeutic drugs [20, 21]. However, in this study, HAIC combined with targeted therapy (anti‐VEGF or anti‐EGFR drugs) didn't show a significantly better survival benefit than HAIC alone. This result, different from some clinical studies, suggests the complexity of CLM treatment response. Previous analyses often attributed this to “drug resistance”. However, analyzing this study's baseline data revealed that combination‐treatment patients had higher tumor burden and treatment‐regimen differences, which confounded efficacy. High tumor burden reflects complex tumor clonal evolution. The combination‐treatment group had a “high‐burden concentration” of intrahepatic lesions: only 3.57% had ≤ 3 lesions, while 53.57% had 4–7 and 42.86% had ≥ 8, compared to 38.00% and 32.00% in the HAIC‐alone group. Also, 60.71% of lesions were 4–5 cm, higher than 30.00% in the HAIC‐alone group. These lesions are prone to polyclonal mutations. Single‐cell sequencing shows that in > 4 cm CLM lesions, RAS/BRAF mutant sub‐clones are 2–3 times more common, with PI3K/AKT pathway activation. The combination‐treatment group had a significantly higher FOLFOX regimen use (71.43% vs. 54.00%, *p* = 0.038). Despite being a classic CLM treatment, high‐burden lesions may be less sensitive to chemotherapy due to DNA damage‐repair gene up‐regulation, weakening the “chemotherapy + targeted therapy” synergy. High tumor burden also enriches CSCs, exacerbating drug resistance. While HAIC can kill proliferating cells, its effect on CSCs is limited. In high‐burden lesions, a hypoxic microenvironment forms due to poor blood supply. HIF‐1α/2α up‐regulation accelerates angiogenesis and induces EMT, weakening anti‐VEGF drug efficacy [22, 23, 24].

Complications of HAIC could be divided into catheter‐related complications and toxicity related to chemotherapy administration. Catheter‐related complications included catheter migration, occlusion, hepatic arterial occlusion, extrahepatic perfusion, or catheter/port related infection, which was consistently reported in approximately 10%–20% of patients in the literature [25]. Moreover, the incidence of complications gradually decreased as the surgeon's experience increased. Our patients had fewer related adverse reactions, which may be related to the routine use of intra‐arterial dexamethasone during surgery [26].

Several limitations should be noted. Given that this is a retrospective study, the patient population within it is highly heterogeneous, and there are substantial differences in the treatment regimens they received. These factors may exert an impact on the treatment outcomes. Although variables such as tumor number and size were accounted for, and a multivariate analysis was conducted, unidentified confounding factors and the small sample size may have weakened the model's ability to adjust for confounders. Therefore, well‐designed prospective studies are needed to evaluate the efficacy of HAIC in a larger sample population, combining molecular targeted therapy with immune checkpoint inhibitors in patients with pMMR/MSS CRLM who are refractory to standard treatments.

In summary, this study provides the real‐world evidence that HAIC is an effective and tolerable treatment in pMMR/MSS patients with CRLM who are refractory to standard treatments. However, the need to combine HAIC with targeted therapy remains to be further confirmed.

## Author Contributions

**Yawei Li:** conceptualization (equal), writing – review and editing (lead). **Junqing Xi:** data curation (equal). **Xiaoyu Huang:** methodology (equal). **Yingen Luo:** formal analysis (equal). **Xiao Li:** project administration (equal), writing – review and editing (equal). **Xiaowu Zhang:** project administration (lead), writing – review and editing (equal).

## Funding

This study was supported by the National Natural Science Foundation of China (Grant No. 82330061), the CAMS Innovation Fund for Medical Sciences (CIFMS) (Grant No: 2024‐I2M‐C&T‐B‐073), and the CAMS Innovation Fund for Medical Science (CIFMS) to Xiao Li (Grant No. 2023‐I2M‐C&T‐B‐085).

## Ethics Statement

In line with the 1975 Declaration of Helsinki, this retrospective study was carried out. The requirement for informed consent was waived by the Independent Ethics Committee of the Cancer Hospital of the Chinese Academy of Medical Sciences and Peking Union Medical College (IRB approval number: 24/151‐4431).

## Consent

The final manuscript was reviewed and approved by all authors.

## Conflicts of Interest

The authors declare no conflicts of interest.

## Data Availability

The corresponding authors can provide data upon a reasonable request.
